# Crops and rising atmospheric CO_2_: friends or foes?

**DOI:** 10.1098/rstb.2024.0230

**Published:** 2025-05-29

**Authors:** Elizabeth A. Ainsworth, Alvaro Sanz-Saez, Courtney P. Leisner

**Affiliations:** ^1^ Crop Sciences and Plant Biology, University of Illinois at Urbana-Champaign, Urbana, IL 61801, USA; ^2^ Auburn University System, Auburn, AL 36849, USA; ^3^ Virginia Tech, Blacksburg, VA 24061, USA

**Keywords:** acclimation, biofortification, climate change, photosynthesis, nutrients, roots

## Abstract

Rising atmospheric carbon dioxide concentration ([CO_2_]) is a ubiquitous global change with direct and indirect impacts on crops. The increase in atmospheric [CO_2_] since the industrial revolution has stimulated photosynthesis in crops and reduced stomatal conductance and canopy transpiration. These physiological changes result in a “CO_2_ fertilization effect” contributing to greater crop yields. However, CO_2_ is a greenhouse gas and has been the major contributor to increased radiative forcing and warmer global temperatures, resulting in more extreme weather events, with negative consequences for crop production. While the benefits of rising [CO_2_] have stimulated productivity to date, they may soon be outweighed by the challenges of rising temperatures and altered precipitation on plant productivity. Rising atmospheric [CO_2_] also reduces the nutritional value of crops, reducing protein content and the concentration of key micronutrients. Distinct physiological mechanisms contribute to changes in crop nutritional value at elevated [CO_2_], but there is potential to harness genetic diversity in nutrient content and for biofortification to counteract the negative impacts of rising [CO_2_] on crop quality. Crop improvement strategies that both adapt crops to future environments and mitigate the negative environmental impacts of agriculture are critical to ensuring future agricultural and nutritional sustainability.

This article is part of the theme issue ‘Crops under stress: can we mitigate the impacts of climate change on agriculture and launch the ‘Resilience Revolution’?’.

## 1. Introduction

**Figure 1.**
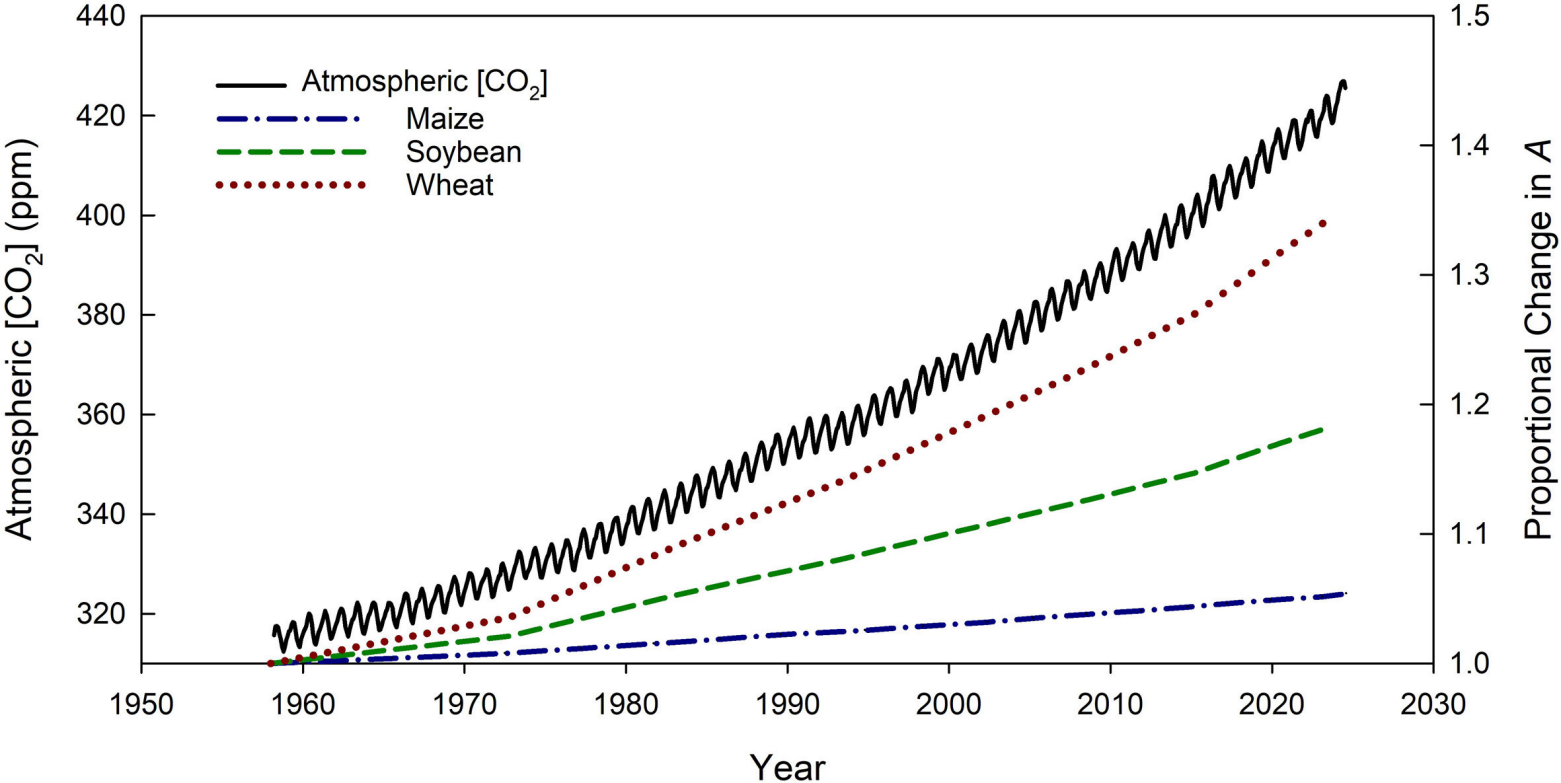
Monthly mean atmospheric carbon dioxide concentration ([CO_2_]) measured on Mauna Loa [1] (solid black line). The dashed and dottes lines indicate the modelled proportional increase in photosynthesis (*A*) in wheat (dotted line), soybean (dashed line) and maize (dotted and dashed line) as atmospheric [CO_2_] has increased from 1957 to 2024.

The concentration of carbon dioxide ([CO_2_]) in the atmosphere has increased by 51% since the Industrial Revolution and by more than 100 ppm since long-term monitoring was started by C. David Keeling at the Scripps Institution of Oceanography in 1958 at Mauna Loa [1] (figure 1). From the 1960s to the 2010s, the decadal mean growth rate in atmospheric [CO_2_] increased from approximately 0.8 to 2.4 ppm yr^−1^ [1,2]. This increase in atmospheric [CO_2_] has been the primary driver of rising global temperatures and more extreme precipitation patterns [3], which reduce crop productivity and exacerbate the negative environmental impacts of agricultural systems [4]. Unlike rising temperatures and changing precipitation patterns, which are spatially variable and temporally inconsistent, CO_2_ is well mixed in the atmosphere, and concentrations are increasing everywhere on the planet. Therefore, understanding how rising [CO_2_] impacts plants is foundational to predicting future crop productivity and interpreting the interactive effects of other aspects of global change. In this paper, we consider if rising [CO_2_] is a friend or a foe for crops, i.e. if rising [CO_2_] is largely beneficial for crop productivity and nutritional quality or if it is detrimental. We start with the photosynthetic basis for this question and consider past and future expected changes in atmospheric [CO_2_]. We then consider the effects of elevated [CO_2_] on crop nutritional quality, the mechanisms contributing to nutrient decline, and identify strategies for maximizing the potential benefits of rising [CO_2_] in the future.

## 2. The response of photosynthesis to increasing atmospheric CO_2_ concentration

**Figure 2.**
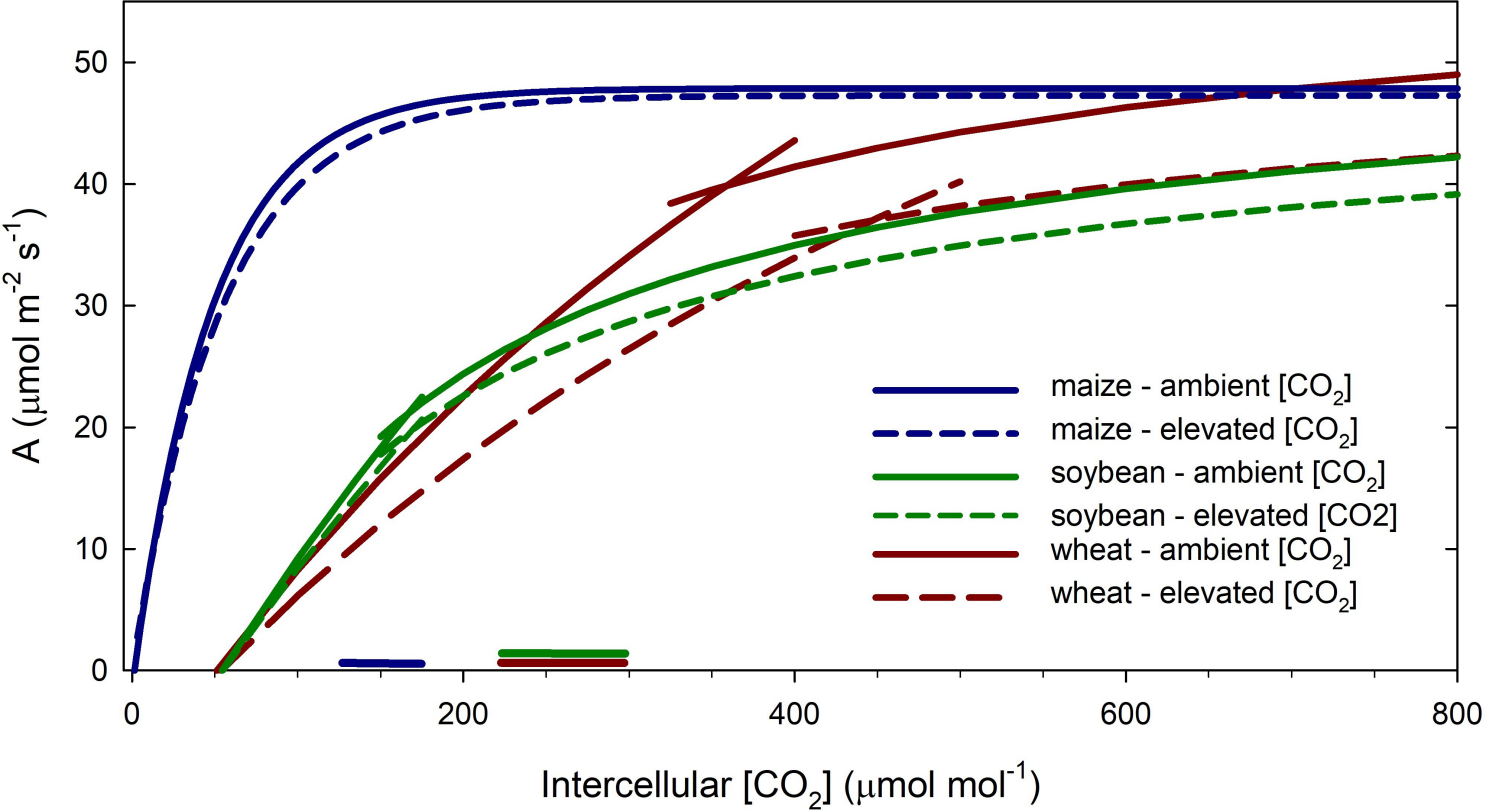
The response of net CO_2_ assimilation rate (*A*) to intercellular [CO_2_] for C3 crops, soybean and wheat, and a C4 crop, maize, grown at ambient (solid lines) and elevated [CO_2_] (dashed lines). Average maximum photosynthetic capacity for soybean and maize was measured in plants grown at the Soybean Free Air [CO_2_] Enrichment (SoyFACE) facility in 2023 and 2024, respectively. The photosynthetic capacity of soybean was measured at 27.5°C. Averaged maximum Rubisco carboxylation capacity (*V*_cmax_) was 194.6 μmol m^−2^ s^−1^ in ambient [CO_2_] and 179.7 μmol m^−2^ s^−1^ in elevated [CO_2_]. The maximum electron transport capacity (*J*_max_) was 260.0 μmol m^−2^ s^−1^ in ambient [CO_2_] and 240.6 μmol m^−2^ s^−1^ in elevated [CO_2_]. The photosynthetic capacity of maize was measured at 28°C. The maximum apparent rate of PEPc activity (*V*_pmax_) was 120 μmol m^−2^ s^−1^, and the CO_2_-saturated photosynthetic rate (*V*_max_) was 48 μmol m^−2^ s^−1^. Wheat photosynthetic capacity was modelled for plants grown at ambient (solid lines) and elevated [CO_2_] (dashed lines) at the T‐FACE experiment in Kangbo village, China [11]. *V*_cmax_ at 25°C was 144 and 113 μmol m^−2^ s^−1^ in ambient and elevated [CO_2_]; *J*_max_ was 314 and 265 μmol m^−2^ s^−1^ in ambient and elevated [CO_2_]. The horizontal lines on the *x*-axis represent the increase in intercellular [CO_2_] for C4 maize (blue) and C3 wheat/soybean (maroon/green) associated with the increase in atmospheric [CO_2_] from 314 ppm in 1958 to 425 ppm in 2024. The relative increases in *A* associated with those increases in intercellular [CO_2_] are plotted in figure 1.

CO_2_ is the substrate for photosynthesis and its increase in the atmosphere stimulates leaf-level rates of CO_2_ assimilation in plants [5–7]. Increased atmospheric [CO_2_] also reduces stomatal conductance in C_3_ and C_4_ plants [6], which typically results in lower canopy transpiration [8]. The response of photosynthesis (*A*) to increasing intercellular [CO_2_] (*c*_i_) provides a theoretical basis for considering how recent increases in atmospheric [CO_2_] may have contributed to increased crop production. In C_3_ crops like rice, wheat and soybean, photosynthesis is limited by the carboxylation capacity of ribulose-1,5-bisphosphate (RuBP) carboxylase/oxygenase (Rubisco) at low intercellular [CO_2_] and at high light [9,10]. Therefore, increasing atmospheric [CO_2_] increases the rate of Rubisco carboxylation and competitively inhibits the oxygenation reaction, resulting in greater rates of *A* as *c*_i_ increases (figure 2). As Rubisco becomes CO_2_-aturated, the regeneration of RuBP limits the rate of photosynthesis, illustrated by the inflection point in the *A*/*c*_i_ curve (figure 2). Increasing *c*_i_ continues to result in greater *A* because of inhibition of the oxygenation reaction, although the increase in *A* is less than in the initial part of the curve [12]. At very high *c*_i_ or conditions where sink limitation can feed back on photosynthesis, triose phosphate utilization (TPU) can limit *A* [13–15]. For simplicity and because the intercellular [CO_2_] for most crops under high light conditions is currently below 300 ppm, we do not consider TPU limitation in figure 2. In C_4_ species like maize, sorghum and sugarcane, CO_2_ is initially fixed by phosphoenolpyruvate (PEP) carboxylase (PEPc) into a C_4_ acid, which is then decarboxylated in bundle sheath cells where Rubisco is located [16]. This CO_2_-concentrating mechanism limits the oxygenation reaction, and consequently, photosynthesis in C_4_ species saturates at a much lower intercellular [CO_2_] compared with C_3_ species (figure 2).

The photosynthetic response of plants to increasing [CO_2_] suggests that rising [CO_2_] is a ‘friend’ or beneficial for productivity (figure 1). In addition to increasing rates of photosynthesis, elevated [CO_2_] also reduces stomatal conductance in plants, enhancing their water use efficiency [6]. This ‘CO_2_ fertilization effect’ has likely contributed to the ability of terrestrial ecosystems to continue to serve as an important sink for increasing anthropogenic CO_2_ emissions [2]. For crops, leaf-level photosynthetic capacity has not dramatically changed between the 1960s and today [17–19]. Therefore, we can use the *A*/*c*_i_ curve to approximate how much the change in atmospheric [CO_2_] since monitoring began on Mauna Loa has increased light-saturated rates of photosynthesis of key crops. We used recently measured values of photosynthetic capacity of soybean and maize grown in central Illinois at the soybean free air CO_2_ enrichment (SoyFACE) experiment [20] and wheat grown in China [11] and assumed the ratio of *c*_i_ to atmospheric [CO_2_] was 0.7 for C3 crops and 0.4 for C4 crops. We then estimated that, from 1960 to 2024, light-saturated photosynthesis increased by approximately 5% for maize, approximately 18% for soybean and approximately 35% for wheat (figure 1). Wheat showed greater stimulation compared with soybean because photosynthetic rates were limited by Rubisco carboxylation in wheat while soybean photosynthetic rates were limited by RubP regeneration (figure 2). Of course, even within a species, photosynthetic capacity varies with environment and crop growth stage [19,21], so our estimates represent a snapshot in time. Still, the estimates provide a general range of stimulation of photosynthesis for C3 and C4 crops associated with increases in atmospheric [CO_2_] over the past few decades. Attributing this CO_2_-induced stimulation in photosynthesis to recent increases in crop yield is complicated by correlations with improved crop varieties, management practices and other technologies [22]. Still, studies have partially attributed the increase C_3_ crop yields and net primary productivity of global cropland ecosystems to rising [CO_2_] [23,24]. In regions where crop yield gains have stalled, studies suggest that recent increases in [CO_2_] have prevented further crop yield losses, counteracting the negative impacts of rising temperature and increased water stress [25]. Thus, to date, studies have largely reported a benefit of rising [CO_2_] on crop productivity.

As global temperatures climb and more extreme heat waves, droughts and floods challenge agricultural productivity, atmospheric [CO_2_] may be less likely to counteract the negative impacts of climate change. This is evident from free air CO_2_ enrichment (FACE) experiments where crops are grown under future high [CO_2_] in open field conditions. It was long hypothesized that the combination of reduced stomatal conductance and increased leaf-level photosynthesis would lessen drought stress in a future high-[CO_2_] atmosphere [26–28]. Unfortunately, consistent experimental support for this hypothesis is lacking [29]. Early season stimulation in biomass at elevated [CO_2_] more than offset lower stomatal conductance in crops, resulting in greater depletion of soil moisture, instead of water savings [30,31]. Wheat tiller survival and grain filling was also reduced by elevated [CO_2_] under dry conditions [32]. The CO_2_ fertilization effect was also significantly reduced by growth at elevated temperatures in FACE experiments, again suggesting that high atmospheric [CO_2_] may not offset the negative impacts of global warming [29]. Thus, it may be that, to date, elevated [CO_2_] and the resultant climate changes have had net positive effects on agroecosystem productivity, but as climate change intensifies, there will be a shift towards net negative effects as has been suggested for natural ecosystems [33].

## 3. Photosynthetic acclimation to elevated atmospheric CO_2_ concentration

C3 crops often decrease investment in Rubisco content and other photosynthetic proteins when grown at elevated [CO_2_], termed photosynthetic acclimation or downregulation [12,27,34,35]. Because C4 photosynthesis is saturated at current atmospheric [CO_2_], acclimation of photosynthetic capacity to elevated [CO_2_] has not been measured in C4 crops in the absence of drought stress [26,36]. In C3 plants, photosynthetic acclimation is common under drought and high-temperature conditions, as well as in the absence of those stresses [35]. The change in photosynthetic protein investment in C3 crops is apparent in the *A*/*c*_i_ curve as a reduction in the initial slope where the carboxylation rate of Rubisco limits *A* and in the asymptote where RuBP regeneration limits *A* (illustrated as the dashed lines in figure 2). As a result of lower investment in Rubisco and other photosynthetic proteins, plants grown in elevated [CO_2_] have lower *A* at a given [CO_2_] compared with plants grown at ambient [CO_2_] [7,12,37]. However, acclimation typically does not eliminate stimulation of photosynthesis when measured at the [CO_2_] in which plants were grown, and increased light-saturated photosynthetic rate at growth [CO_2_] is one of the most ubiquitous responses of C3 crops to elevated [CO_2_] [6].

Photosynthetic acclimation at elevated [CO_2_] is more pronounced when environmental, genetic and/or developmental factors limit sink strength [7,27,37]. For example, non-nodulating soybeans, tobacco with low leaf area and wheat with reduced tillering capacity showed significant downregulation of photosynthetic capacity at elevated [CO_2_] [38–40]. Acclimation in rice to elevated [CO_2_] was greatest during the latter part of the growing season when stimulation of biomass and enhancement of leaf area were less than in the earlier parts of the growing season [37]. These conditions are associated with low sink strength, and, not surprisingly, increasing sink strength has been proposed as a key mechanism for minimizing photosynthetic acclimation [41,42] and maximizing crop responses to elevated [CO_2_] [29]. The accumulation of foliar carbohydrates at elevated [CO_2_] has long been hypothesized to be associated with the downregulation of photosynthetic capacity [43–45], resulting from insufficient sink capacity [46]. In wheat, adaptive sink plasticity may have been selected against during crop domestication, which may limit the response of wheat to elevated [CO_2_]. Enhancing root growth, improving vegetative sink plasticity and enhancing reproductive sinks are hypothesized to be key traits needed to maximize future wheat yields [42]. However, enhancing vegetative growth may not always be beneficial at elevated [CO_2_] [30]. In soybean, the optimal leaf area index for maximizing yields at elevated [CO_2_] is lower than the leaf area index produced by modern cultivars, suggesting that reducing leaf area, not increasing it, might improve yields, especially if harvest index can be maintained or increased [47]. Of course, carbon sources and sinks also must be balanced with nitrogen and other nutrient sources and sinks for optimal growth, and acclimation of photosynthesis at elevated [CO_2_] is evidence for feedback between sources and sinks [41].

Perhaps photosynthetic acclimation can be thought of as detrimental and limiting the yield response of crops to elevated [CO_2_], but reducing the investment in Rubisco and other photosynthetic proteins is also important for optimizing photosynthetic capacity with growth and nitrogen use efficiency [7,48–50]. Lower photosynthetic demand for Rubisco at elevated [CO_2_] reduces N content in leaves [51]. Optimality theory suggests that acclimation of photosynthesis is needed to maintain high rates of photosynthesis at the lowest possible nutrient use [49]. However, lower N content in crop leaves could also provide less N for filling fruits and seeds [52]. Thus, acclimation of photosynthesis can be a ‘friend’ in terms of optimizing resources in natural ecosystems but a ‘foe’ for maximizing the yield potential and nutritional quality of crops produced in elevated [CO_2_].

## 4. Effects of elevated CO_2_ on nutritional quality

The positive ‘CO_2_ fertilization’ effect on crop yield is complicated by a decrease in many nutrients in elevated [CO_2_] [53–59]. An analysis of 130 varieties of plants found that elevated [CO_2_] decreased the concentration of 25 important minerals by 8% on average, and the carbohydrate to mineral ratio was increased in these plants [56]. While seed germination was not compromised in seeds produced from crops grown at elevated [CO_2_] [60], the nutritional value of seeds was decreased in elevated [CO_2_]. Myers *et al*. [57] reported that elevated [CO_2_] decreased zinc (Zn) and iron (Fe) by 9.3 and 5.2%, respectively, in C3 grasses and legumes. The impact of elevated [CO_2_] on Zn and Fe has the potential to decrease global availability of these nutrients, which could disproportionately affect countries with high levels of human malnourishment [58,61–65]. However, crop varieties greatly vary in nutrient content, offering hope that this challenge can be addressed through selection [29].

Elevated [CO_2_] alters protein, oil and carbohydrate content in commodity and food crops [53,66–68]. Total protein concentration decreased by 10–15% in elevated versus ambient [CO_2_] for barley, rice, wheat, soybean and potato [59,66,69]. This is likely due to the observed decrease in nitrogen (N) content in plants grown in elevated [CO_2_] [56,58,70,71] (see hypothesized mechanisms below). Furthermore, elevated [CO_2_] significantly decreased vitamin B by approximately 12–30% in rice [59], and a recent meta-analysis found plant carotenoid concentrations were also decreased in elevated [CO_2_] [72]. The nutritional quality of C4 plants is expected to be less affected than that of C3 plants [56,57,73].

Elevated [CO_2_] increased total phenolic content and total antioxidant capacity in some vegetable crops [53,70], as well as vitamin E content in rice [59]. This is important as phenolic compounds, along with phytic acid, act as major inhibitors of Fe absorption and thus inhibit bioavailability [74]. Phenolic compounds can also interact with proteins to alter their physicochemical properties, which can change their solubility and digestibility [75]. Phenolic compounds have also been shown to both increase and decrease absorption and bioavailability of Zn [76].

Many factors might lead to the variation in nutritional responses of crops to elevated [CO_2_]. Certainly, different soils and environments impact experimental results, and there is also significant genotypic variation in plant responses to elevated [CO_2_]. For example, studies of soybean have reported differences in response to elevated [CO_2_] in terms of seed nutrient content and yield [57,77–79]. Continued research on understanding the mechanism associated with changes in nutrient content in edible tissues in elevated [CO_2_] is needed to ensure future food and nutritional security goals.

Several hypothesized mechanisms have been proposed to describe the decreased nutrient content observed in C_3_ plants grown under elevated [CO_2_]. The first is a function of *lower transpiration*. Decreased mineral content in seeds under elevated [CO_2_] could be a consequence of decreased transpiration, which reduces the transfer of nutrients from roots to shoots [80]. Minerals travel as dissolved molecules in the xylem and therefore depend on the transpiration stream to pull them from the roots to aboveground biomass. Under elevated [CO_2_], stomatal conductance decreases [7], which results in reduced canopy transpiration [8] and mass flow of nutrients to leaves [80–82]. A second mechanism is *downregulation of photosynthesis*. As described above, plants decrease investment in Rubisco and other photosynthetic enzymes at elevated [CO_2_], resulting in less N available for translocation to developing seeds. Less investment in photosynthetic proteins could also *alter requirements for minerals owing to changes in enzyme/organic complex requirements* [80]. For example, a decrease in magnesium (Mg) uptake might occur if there were decreased demand for Rubisco and/or chlorophyll, as Mg is required for both. Furthermore, the ratio of manganese (Mn) to Mg in leaves may also play a role in acclimation to elevated [CO_2_] levels [82]. *Mineral dilution* is perhaps the most intuitive mechanism contributing to decreased nutrient content at elevated [CO_2_]. Greater carbohydrate production and content at elevated [CO_2_] dilutes mineral nutrient concentration in seeds and other organs [62,69,83]. There is also evidence that *inhibition of nitrate assimilation owing to decreased reducing power* occurs at elevated [CO_2_]. Lower rates of photorespiration at elevated [CO_2_] could decrease the amount of reducing power to drive the reduction of $NO_{3}^{-}$ to $NH_{4}^{+}$ by nitrate reductase and nitrite reductase (NiR) [84–86]. Increased photosynthesis under elevated [CO_2_] could also decrease the availability of reduced ferredoxin needed for NiR [87]. These decreases in reducing power would thereby decrease the overall N concentration and ultimately protein content in plants [71,88]. Finally, *reduced mineral absorption and altered root architecture* along with *altered expression of transporters* could contribute to lower nutrient content in elevated [CO_2_]. Previous work has suggested that a reduction in mineral absorption in root tissue occurs under elevated [CO_2_] [7], and elevated [CO_2_] can affect root architecture and physiology [81,89]. Zn and Fe transporters can also be decreased in root, stem and leaf tissue of plants grown under elevated [CO_2_] [82], which may influence the flux of these nutrients in a mineral- and organ-specific manner.

The literature provides evidence for and against each of these mechanisms. To date, no work has identified a universal mechanism that is consistent across all nutrients and all crops and environments. Additionally, more work is needed to understand how other environmental factors will interact with rising [CO_2_] to impact nutritional quality. For example, experiments investigating concurrent increases in [CO_2_] and warming have reported that warming can either ameliorate or worsen the impacts of elevated [CO_2_] on seed or grain quality [67,90–94]. Köhler *et al*. [91] found that elevated temperature (3.5°C above ambient) ameliorated the negative impacts of elevated [CO_2_] (600 ppm) on Zn and Fe content in soybeans. Elevated temperature also decreased seed protein concentration and increased oil concentration regardless of atmospheric [CO_2_], though the effect was dependent on canopy position of the seeds [91]. Xu *et al*. [92] also found that elevated temperature decreased soybean protein content, but only in ambient [CO_2_], not in elevated [CO_2_] (800 ppm). Palacios *et al*. [95] found no effects of elevated [CO_2_] or temperature on protein and oil content, while Thomas *et al*. [67] found the mean oil concentration of mature soybean seeds was highest at 32/22°C and decreased with further increases in temperature, though there was no significant effect of elevated [CO_2_] (700 ppm) on mean oil concentration. These studies were all done with different cultivars and different soils, and clearly more work is needed to fully understand and predict how the combination of rising [CO_2_] and warming will impact soybean quality.

Work done in rice grown at elevated [CO_2_] (ambient + 200 or 300 ppm) and elevated temperature (+4°C) found decreased protein concentration in the grain, with no interactive effects between elevated [CO_2_] and temperature [90]. In contrast, Wang *et al*. [93] reported a reduction in protein concentrations in both rice and wheat grown in elevated [CO_2_] (500 ppm), but no change in protein in response to canopy warming treatments (+2°C), indicating a significant CO_2_ by temperature interaction. Macro-element concentrations were also unaffected by growth of rice at elevated [CO_2_] (500 ppm) and warming conditions (+2°C) [94], but there was an increase in the concentration of heavy metals. This has the potential to increase the likelihood of heavy metal toxicity in rice grains in the future. Whether some or all the proposed mechanisms described above are impacting nutritional quality of grains at elevated [CO_2_] and elevated temperature requires more research. Future efforts to study how elevated [CO_2_] impacts nutritional quality of crops should expand into a more diverse set of crops and greater environmental and edaphic conditions.

## 5. Nutrient uptake through roots at elevated atmospheric CO_2_ concentration

While it is known that elevated [CO_2_] increases plant photosynthesis and therefore above-ground biomass accumulation [79], the effect of elevated [CO_2_] on roots is less well understood owing to the difficulty of studying root development [96,97]. Controlled and open field experiments in agricultural commodities, pastures and trees observed that elevated [CO_2_] tends to stimulate root growth by increasing root length, diameter and complexity, and rooting depth [89,98–102]. However, other studies found that elevated [CO_2_] did not stimulate root growth in some species and/or under N-limiting conditions [89,98,103].

In tall fescue, the positive effect of elevated [CO_2_] on root growth under moderate N content was related to increased concentrations of indole-acetic acid (IAA), an auxin [103,104]. and isopentenyl adenosine (iPA), a cytokinin [103]. Elevated [CO_2_] increased the expression of genes related with the accumulation of IAA, such as *FaYUCCA11* [103], *YUCCA8* and *YUCCA9* [104], which are related to growth of secondary roots [103]. Elevated [CO_2_] also increased the expression of *FaIPT8*, which increases the amount of iPA and downregulates the gene *FaCKX1*, which encodes a cytokinin oxidase responsible for iPA degradation [103]. These changes in expression under elevated [CO_2_] increase accumulation of cytokinins [103] and stimulate root growth [105]. This hormonal interaction may be responsible for the increase in root/shoot ratio observed under elevated [CO_2_] [89].

Increased partitioning of biomass to roots accompanied by greater root length, number of root tips and deeper roots [89,99,100] could improve soil exploration and increase nutrient and water uptake. Uddin *et al*. [102] reported that elevated [CO_2_] increased water uptake and drought tolerance of wheat because of greater root length deeper in the soil where water was available even under drought conditions. Similarly, the canola cultivar ‘Thumper’ produced more biomass and yield under drought and elevated [CO_2_] owing to more abundant roots in the deeper layers of the soil. Although few field studies have investigated root responses to elevated [CO_2_] in detail, nutrient yield measured as nutrient per plant or per area is often increased in elevated [CO_2_] [106–108]. Increased root biomass and area would support the increased uptake needed for greater nutrient yields.

Although it has been demonstrated that elevated [CO_2_] may increase the capacity of roots to explore and extract more nutrients and water from the soil, there may be a hidden foe. Increased root growth at elevated [CO_2_] was accompanied by a reduction in the stele area [94], which significantly reduced xylem (20–40%) in comparison with roots grown at ambient [CO_2_] [99,100]. This reduction in xylem area may limit stomatal conductance and transpiration at elevated [CO_2_] [6,78,100,106] and contribute to decreased nutrient concentration at elevated [CO_2_] [71]. A decrease in xylem area could also contribute to impaired N transport at elevated [CO_2_] [99,100] along with the downregulation of N transport genes from the root to the shoot [82]. However, the effects of elevated [CO_2_] on the expression of transport genes differ across species and developmental stages [71,82,108,109], and more research is needed to fully understand the downstream impacts of altered root anatomy and gene expression at elevated [CO_2_].

In rice, it has been observed that the elevated [CO_2_]-mediated reduction of Fe and Zn in the seed may be caused by the downregulated expressions of genes related to Zn and Fe transport [110]. Elevated [CO_2_] reduced the expression of the zinc transporter 11 precursor (ZRT/IRT-like protein 11) gene involved in Zn transport, and the expression of *OsZIP3,* a zinc transporter 1 precursor (ZRT/IRT-like protein 1) involved in the transport of Zn and Fe [110]. Similarly, the expression of *OsZIP5* involved in the transport of Mn, Fe, Cu and Zn was reduced at elevated [CO_2_] [110]. Assuming these changes in gene expression are consistent across crops, then key targets for ameliorating the reduction of essential nutrients such as Fe and Zn at elevated [CO_2_] are in hand [56,57,71]. Independent of studies of the effects of elevated [CO_2_] on plant nutrient concentration, scientists around the world have created international consortiums like the Food and Agriculture Organization (FAO)'s ‘HarvestPlus’ to increase essential mineral and vitamin concentrations in staple foods through natural and metabolic biofortification to solve the hidden hunger problem [111–113].

## 6. Natural variation and transgenic biofortification as a tool to increase essential minerals in staple foods at elevated atmospheric CO_2_ concentration

**Figure 3.**
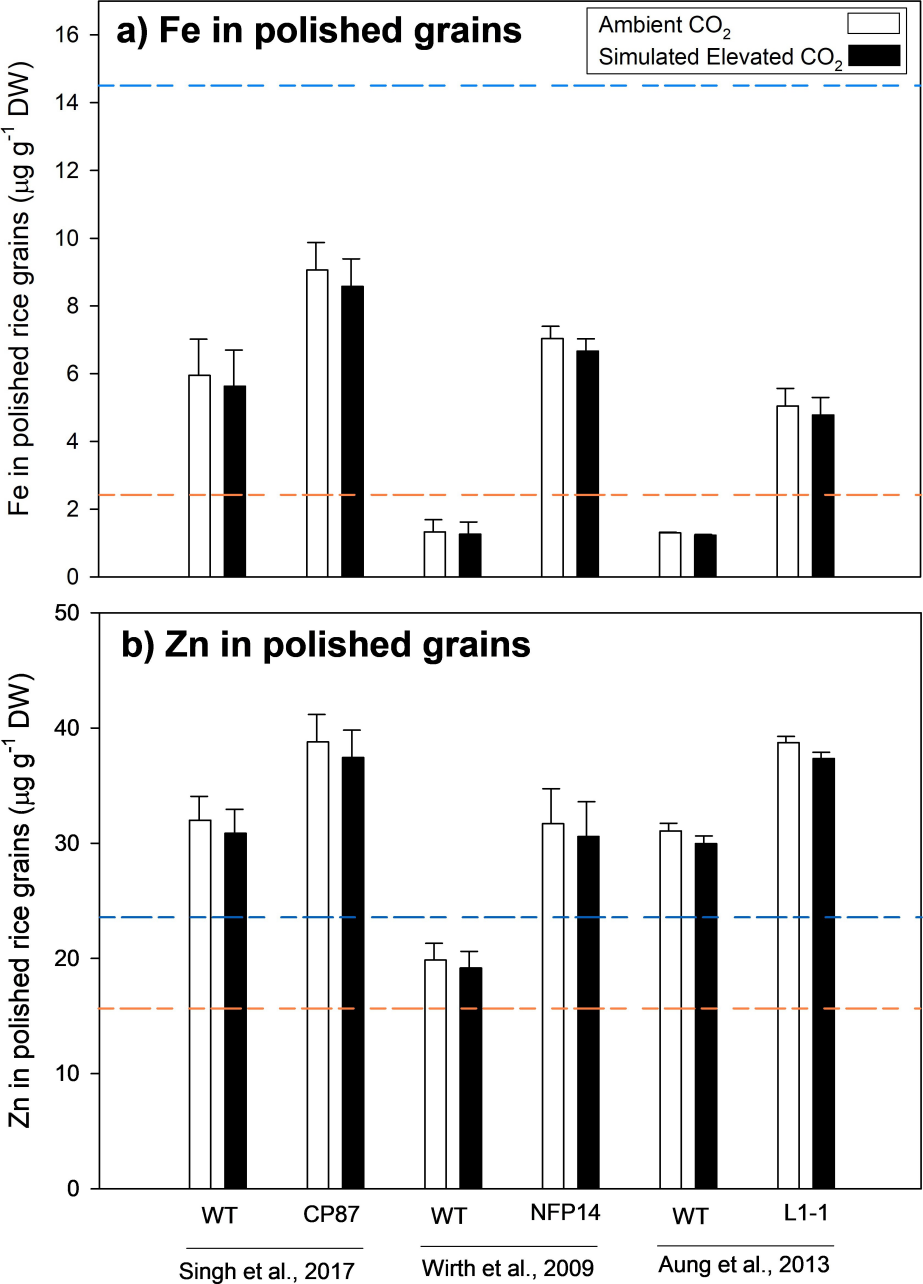
Iron (Fe) (*a*) and zinc (Zn) (*b*) concentration in rice polished grains from three transgenic biofortification experiments [111,115,118]. White bars represent the average values of Fe and Zn in biofortified transgenic plants (CP87, NFP14, L1-1) grown under ambient [CO_2_]. Black bars represent the predicted Zn and Fe concentration at elevated [CO_2_], based on the measured reduction in each mineral from [57]. The orange line represents the baseline Fe and Zn concentration measured in current commercial varieties, while the blue line represents the concentration needed to reach the target daily requirements for each mineral [117]. DW, dry weight.

Variation in seed mineral content of main staple foods such as common bean, rice, pearl millet and wheat exists among genotypes and can be used to breed high-yielding cultivars with high nutrient concentrations [112–114]. For example, scientists involved in the FAO programme HarvestPlus have used cultivar variation in Fe and Zn to increase the seed nutrient concentration almost twofold to provide up to 70–80% of the daily Zn and Fe requirements [113]. Over the last decade, genetically modified crops that capture and transport more Fe and Zn have been produced by over-expression of genes regulating phytosiderophores [111,115,116] and ferritin [111,116]. In polished rice, this approach increased the Zn concentration two to three times above the baseline compared with commercial varieties [117], greatly surpassing the target daily Zn requirement (figure 3) [111,115,116,119]. Biofortification has also increased Fe concentrations by twofold to fivefold, although this has not been enough to reach the breeding target (figure 3). The 3.5% decrease in Zn content commonly measured at elevated [CO_2_] [57] could be more than compensated for by switching to Zn-biofortified varieties (figure 3). However, the 5.2% decrease of Fe concentration under elevated [CO_2_] would make it more difficult to reach the target daily Fe requirement [119] (figure 3). The strategy of using biofortified crops to combat rising [CO_2_] is promising, but more research is needed to test biofortified rice and other crops in field experiments under elevated [CO_2_].

## 7. Conclusions and future research directions

A major sustainability goal for plant scientists is to adapt crops to future environments while increasing the sustainability of cropping systems. There are several research directions that could help ensure that crops continue to benefit from rising [CO_2_]. These include:

— Selection for plasticity in growth, improved sink strength and high harvest index could maximize the yield response to rising [CO_2_] [29].
— A more nuanced and thorough understanding of the environmental and genetic mechanisms underpinning photosynthetic acclimation to elevated [CO_2_] is needed to maintain protein content in edible tissues while improving the nutrient use efficiency and sustainability of future cropping systems [71].
— Selection of cultivars with enhanced root growth and improved root anatomy could have compounding benefits of enhancing productivity at elevated [CO_2_] and sequestering more carbon [119].
— Continued research to improve Zn and Fe content and bioavailability is important, but additional work is needed to understand how other key nutrients important to the human diet such as lithium, selenium, chromium, iodine and fluorine are impacted by rising [CO_2_] [114].
— Further development of crop models and simulations that include nutritional quality could also contribute to improved understanding of the mechanisms by which elevated [CO_2_] may be negatively impacting plant nutritional quality [114,120].

To facilitate this research, engagement and collaborations between experts in human nutrition as well as agricultural and plant scientists are critical [114]. Additionally, across all research avenues, it is imperative that we include a greater variety of crop species, including specialty crops and perennial plants, and diverse growing environments [121] to gain a more complete understanding of how elevated [CO_2_] impacts crop productivity and nutritional quality.

## Data Availability

Our figures use publicly available data and previously published data. The data are already available.
